# Navigating ethical waters: The impact of ethical judgment, norms, and CSR skepticism on CSR engagement and purchasing intention in a breast cancer campaign

**DOI:** 10.1371/journal.pone.0352994

**Published:** 2026-07-02

**Authors:** Yang Cheng, Yangzhi (Nicole) Jiang, Qizhao Zhang

**Affiliations:** 1 Department of Communication, North Carolina State University, Raleigh, North Carolina, United States of America; 2 School of Communication, Northern Arizona University, Flagstaff, Arizona, United States of America; 3 Department of Organizational Sciences and Communication, The George Washington U, Washington, District of Columbia, United States of America; Universitat Jaume I, SPAIN

## Abstract

Corporate social responsibility (CSR) campaigns play a crucial role in shaping consumer perceptions and behavior. This study examines Delta Air Lines’ CSR campaign centered on breast cancer awareness, focusing on how ethical judgment and subjective norms are associated with CSR skepticism and how such skepticism, in turn, relates to CSR engagement and ethical purchasing intention. Drawing on the theory of reasoned action (TRA), a quantitative survey was conducted with 787 Delta Air Lines customers, all of whom provided informed consent prior to participation. The data, analyzed between August 1–7, 2024 using structural equation modeling, show that ethical judgment and subjective norms are associated with lower levels of CSR skepticism. In turn, CSR skepticism is negatively related to both CSR engagement and ethical purchasing intention, while higher levels of CSR engagement are associated with greater ethical purchasing intention. These findings provide insight into the role of ethical and social factors in shaping consumer responses to CSR initiatives and highlight the importance of fostering engagement to enhance the effectiveness of CSR campaigns and promote favorable consumer outcomes.

## Introduction

In the contemporary business landscape, corporate social responsibility (CSR) has become increasingly important for business legitimacy, sustainability, and profitability [1–2]. Companies are increasingly engaging in various voluntary activities that extend beyond their profit-driven interests and legal obligations to further societal and environmental goals, such as sustainability, ethical sourcing, community welfare, and social justice [2–6]. Large corporations, including Fortune 500 firms, have substantially increased their investments in CSR initiatives in recent years, reflecting the growing strategic importance of CSR [7]. For example, Delta Air Lines has been actively involved in promoting community well-being through its annual Breast Cancer Awareness Campaign since 2005 [8]. Through its iconic activities, such as the “Breast Cancer One” flight and the SkyMiles Donation program, Delta engages its customers, employees, and other stakeholders in its annual CSR campaign, aiming to raise public awareness of breast cancer, encourage early detection and treatment, and raise funds for breast cancer research [8–9].

Previous CSR research has demonstrated that well-executed CSR campaigns not only generate societal benefits but also yield substantial advantages for companies. Organizations involved in CSR enjoy enhanced brand reputation, increased stakeholder support, and, in some cases, improved financial returns (e.g., [10–13]). Notably, an organization’s CSR initiatives are widely shown to be associated with consumer perceptions and behaviors, as ethically minded consumers tend to reward companies demonstrating a commitment to social responsibility while penalizing those with unethical practices [14]. Over 80% of U.S. consumers expressed willingness to purchase products from companies advocating for social issues they cared about or offering products with social and environmental benefits; nearly nine out of ten consumers reported a tendency to boycott brands perceived as operating unethically [15]. These insights suggest that consumers’ ethical purchasing intentions are closely related to corporate outcomes and may also have broader societal implications. Consequently, the United Nations has identified ethical and sustainable consumption as a major goal in the 2030 Agenda for Sustainable Development [16].

The theory of reasoned action (TRA) [17] has been widely adopted to understand consumer behavior, particularly in the context of ethical consumerism (e.g., [18–20]). According to TRA, consumers’ ethical consumption intentions are shaped by attitudes and perceived social expectations. However, skepticism complicates the relationship between attitude and behavior in the realm of CSR. The tension between the profit maximization of businesses and voluntary social activities, coupled with the prevalence of marketing-oriented campaigns, often generates consumer skepticism toward CSR [21–22]. Consumers may be less likely to support CSR initiatives and the organization when they doubt the altruism and authenticity of CSR campaigns [23]. This highlights the importance of moral evaluation in shaping consumers’ perceptions and responses, as authenticity and altruism reflect the moral values and positions of the organization [24–25]. Although the existing literature has identified a variety of factors influencing CSR skepticism, such as fit, attributions, and commitment (e.g., [26–28]), it leaves the ethical perspective on CSR skepticism underexplored.

To address this research gap, this study pays particular attention to the ethical root of skepticism toward socially responsible initiatives and incorporates ethical judgment, referring to an individual’s attitudinal evaluation of the morality of an organization’s CSR efforts [13], in the CSR context to provide a more comprehensive understanding of skepticism and ethical consumption decisions. Specifically, this study adopts Delta’s Breast Cancer Awareness campaign as the study background and proposes that ethical judgment, combined with perceived social pressure to purchase from ethical companies, is associated with lower levels of consumers’ skepticism toward CSR, which is further linked to higher intentions to engage in CSR activities and form ethical purchase intentions. In our research model, CSR skepticism acts as a mediating variable between ethical judgment, as well as subjective norms, and ethical purchase intentions. The findings from this study extend the TRA framework by incorporating ethical judgment and CSR skepticism as central variables, thereby offering a more comprehensive understanding of the cognitive mechanism of consumers’ ethical consumption decisions in the context of CSR. Additionally, this research provides strategic and practical insights for organizations seeking to design CSR campaigns that resonate with ethically conscious consumers while reducing skepticism.

## Literature review

### Ethical consumption behaviors through the reasoned action approach

Ethical consumption refers to “the conscious and deliberate choice to make certain consumption choices due to personal beliefs and values” [29, p. 370]. It includes purchasing ethical products, contributing to social causes, demonstrating preferences for ethical brands, engaging in ethical campaigns, and boycotting unethical brands [19,30,31]. In this study, we view consumer engagement in CSR initiatives and purchasing products from an ethical company as ethical consumption.

In essence, this ethical behavioral pattern reflects consumers’ concern for business ethics and their obligations to the larger society [32]. Ethical consumption is closely tied to sustainability and social responsibility. As consumers increasingly prioritize ethical considerations in their consumption decisions, businesses are compelled to adopt more socially responsible practices [33], and vice versa; the flourishing of socially responsible campaigns in the marketplace motivates more ethical consumption [34]. These, in turn, contribute to a better society. From the perspective of consumer behavior, this study adopts the TRA model to examine how attitudes and perceived social norms influence consumers’ purchasing intentions, providing businesses with practical implications to improve their CSR campaigns.

The TRA, developed by Fishbein and Ajzen [17], offers a robust framework for understanding consumers’ ethical consumption behaviors (e.g., [18,20,35–37]). According to this model, an individual’s behavior is primarily influenced by their intention to take a specific action, which reflects both the likelihood and willingness to engage in that behavior [38]. Behavioral intentions are shaped by individual attitudes and perceived subjective norms regarding the action in question.

**Fig 1:**
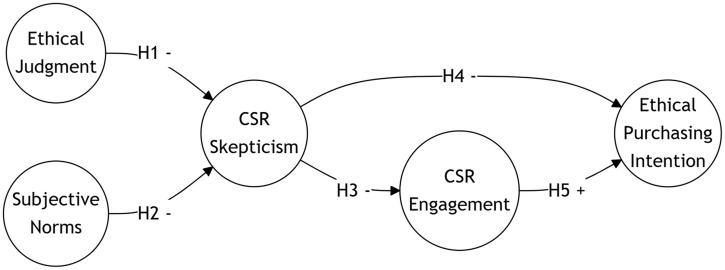
Conceptual model.

To further clarify the conceptual distinctions among the key constructs in this study, ethical judgment reflects consumers’ evaluation of the moral appropriateness of the CSR initiative, whereas CSR skepticism captures doubt about the authenticity and underlying motives of CSR activities. CSR fit refers to the perceived congruence between the company and the CSR initiative, and CSR engagement reflects behavioral responses such as information seeking and participation. Fig 1 illustrates how ethical factors, such as ethical judgment and subjective norms, influence CSR skepticism. This conceptual model also explores the consequences of CSR skepticism, including CSR engagement and ethical purchasing intention. Extensive research has demonstrated that skepticism can significantly undermine consumer responses to CSR initiatives, particularly when consumers perceive self-serving motives and question the sincerity of an organization’s efforts [39–40]. This skepticism about a company’s ethical stance is critical in shaping consumer perceptions and behaviors, highlighting the need for organizations to address these concerns to foster positive engagement with their CSR activities.

### Ethical judgment as attitudinal evaluation

According to Fishbein and Ajzen [17], attitude refers to an individual’s general assessment of engaging in a particular behavior, which is determined by one’s accessible beliefs about the behavioral consequences. These beliefs reflect the individual’s subjective probability that engaging in the behavior will lead to specific outcomes, and the strength of these beliefs directly influences the individual’s attitude [41]. Thus, attitudes are developed through evaluation, in which individuals appraise the characteristics linked to behaviors or objects and determine the appeal of those characteristics based on their existing knowledge and beliefs [41].

In the context of CSR campaigns, consumers primarily assess these activities through an ethical lens because CSR campaigns inherently deal with issues of moral responsibility and corporate citizenship [42–43]. When consumers encounter CSR campaigns, they engage in an attitudinal evaluation of the ethical dimensions of these campaigns. This process, known as ethical judgment, involves deliberative assessments of the actions of an organization that are made concerning moral equity, such as perceived justice, fairness, and sincerity, evaluating the right or wrong of those actions [13,35,44]. It represents an attitudinal response that reflects consumers’ evaluations of whether the CSR activities align with their ethical values and moral standards [45–47].

Ethical judgment, in this sense, can be viewed as a specific type of attitude based on the moral appraisal of an organization’s behaviors [35]. According to Argyriou and Melewar [48], attitudes are formed based on information accessible in memory. In the case of CSR, ethical knowledge and principles play a significant role in shaping consumers’ attitudes. When consumers are exposed to CSR information, such as sustainability reports or social initiatives, they retrieve ethical principles stored in their cognitive frameworks. By comparing the CSR activities with these principles, consumers form an ethical judgment—essentially an attitude that reflects their moral evaluation of the company’s actions.

As ethical consumption, purchasing decisions that reflect the consumers’ commitments and concerns of ethics, is a growing trend in the marketplace [49–50], ethical judgment is essential because it functions as a critical antecedent influencing consumers’ ethical purchasing intentions and behaviors [45–47]. Reinecke and Ansari [51] argued that ethical judgment shapes consumers’ understanding of ethical situations. A favorable ethical judgment, where consumers view CSR initiatives as aligned with their moral values, is linked to favorable attitudes toward the company and an increased likelihood of ethical purchasing willingness (e.g., [52–53]). In contrast, the perception of misalignment between corporate performance and personal moral norms may trigger consumers’ outrage, skepticism, and boycott (e.g., [22,46]).

### Subjective norms

Ethical consumption is not solely a result of consumers’ ethical judgment but also a product of subjective norms [35]. According to TRA, subjective norms reflect the social pressures present in human interactions, which influence individuals to behave or not behave in a certain way [36]. These social pressures stem from the expectations of important referent groups, such as family, friends, or society at large, and shape individual behavioral intentions [38]. As social beings, humans naturally desire to manage their social image and please their important referents [35]. Perceived subjective norms, therefore, shape individuals’ behaviors.

Numerous previous studies have consistently shown that consumers are more inclined to adopt socially responsible consumption patterns when they believe that their significant others or peer groups expect or approve of such behavior [35,54–56]. For example, Robichaud and Yu [57] unearthed that Generation Z exhibited a heightened propensity to procure fair-trade coffee when they perceived an expectation from their family, friends, and other influential individuals. The impact of subjective norms is particularly potent when CSR initiatives resonate with widely embraced societal values, such as sustainability. Golob et al. [58] found that consumers’ environmentally conscious purchasing behavior, such as the preference for products with minimal air pollution, is significantly influenced by their perceived social expectations to select environmentally friendly alternatives. Moreover, external social norms on environmentally friendly consumption could be internalized to shape consumers’ ethical standards, ultimately shaping consumers’ ethical consumption behaviors and contributing to a broader movement toward sustainable and ethical consumption.

### CSR skepticism

The concept of skepticism has received much attention in CSR literature (e.g., [22,23,27]). It is broadly understood as a consumer’s propensity to distrust, doubt, and question CSR-related claims and actions [22,26,28]. This skepticism often arises when consumers perceive a contradiction between a corporation’s profit-driven nature and its purported altruistic CSR activities [59]. Rim and Kim [22] identified skepticism regarding the altruism of CSR campaigns by an organization as the leading cause of unfavorable public responses, such as negative brand attitudes and diminished supportive intentions. In other words, attributing CSR initiatives as genuinely benefiting society or purely business strategies for profit maximization is significantly related to consumers’ perceptions and behaviors regarding CSR. Therefore, for this study, we define CSR skepticism as consumers’ tendency to distrust, doubt, and question a corporation’s motives and the authenticity of its socially responsible actions [60].

Researchers have identified various factors influencing consumers’ skepticism, such as CSR fit, attributions, history, timing of the CSR initiatives, and communication strategies [39]. CSR literature often treats skepticism as a cognitive response triggered by contextual factors (e.g., [28]), meaning it can be mitigated by changing consumers’ surrounding situations. For example, providing consumers with transparent and substantial information about CSR activities, highlighting the public’s relevance to the CSR issue, and underscoring the intrinsic, altruistic motivations of these efforts have been shown to attenuate skepticism [23,60]. Despite these advances, much of the existing literature focuses on how external factors (e.g., CSR communication; [23]) are associated with skepticism, with less attention paid to consumers’ internal cognitive mechanisms. As skepticism inherently involves questioning a company’s ethicality and motives, it has profound ethical implications.

Doubting a company’s authenticity or altruistic motives reflects concerns about its moral values and integrity [24–25]. Recent research underscores the importance of an ethical perspective in mitigating skepticism, suggesting that a broader understanding of CSR skepticism may benefit from an ethical lens, particularly by examining ethical judgment from the consumer’s perspective. For instance, Toti and Romero [61] demonstrated that perceptions of brand ethicality could effectively diminish consumer skepticism toward CSR efforts. Cheng and Shen [62] further highlighted that negative ethical judgments can erode trust in enterprises, suggesting a critical relationship between ethical judgment and skepticism: when consumers form negative ethical judgments, their distrust intensifies, further fueling skepticism toward the company’s actions and motives. Shim and Yang [63] pointed out that in the context of CSR, corporate hypocrisy reflects the public’s moral judgment of corporate philanthropy and prosocial behavior. The perception of hypocrisy can negatively impact a company’s reputation and increase skepticism about its CSR motives. Given this context, it is evident that consumers’ ethical judgments regarding a company’s CSR activities are intricately linked to their skepticism. Thus, it is reasonable to posit H1 below.

**H1:** Ethical judgment is negatively associated with CSR skepticism.

Previous literature indicates that subjective norms also play a crucial role in shaping consumers’ attitudes and behaviors toward CSR initiatives. These norms, defined as perceptions of what important referent groups expect of an individual [37], are significantly related to how people evaluate and engage with CSR activities. For example, Lee et al. [64] explored the effects of Twitter followers and consumer skepticism on support behavior for issues promoted in Twitter-based CSR communication. The findings suggest that when consumers see others endorsing the social causes advocated in these CSR activities, they are more likely to accept the behaviors promoted in social networking site (SNS) campaigns and reduce their skepticism.

Research has shown that social influence can enhance trust in CSR messages. For instance, when consumers observe friends or family endorsing a company’s CSR efforts, they may be less inclined to question the motives behind these initiatives. This aligns with the idea that positive feedback from social circles can foster a sense of collective approval, which diminishes skepticism about a company’s intentions [65]. Moreover, when consumers perceive subjective norms, such as the approval of important others regarding ethical consumption, they are more likely to internalize these values and adopt similar attitudes [57]. This pattern is associated with lower levels of skepticism toward CSR activities, as consumers may feel a sense of obligation to align their behaviors with the expectations of their social group [55].

In summary, subjective norms are significantly associated with lower levels of CSR skepticism and are further linked to more positive perceptions of CSR efforts and greater engagement in socially responsible practices. We thus propose H2.

**H2**: Subjective norms are negatively associated with CSR skepticism.

### CSR engagement

Despite the nascence of the concept of engagement in both marketing and CSR literature, the significance of engaging strategic stakeholders for business performance and success has been identified, particularly in the context of CSR [66–68]. From the organizational perspective, Greenwood [69] defines stakeholder engagement as an organization’s effort to actively involve stakeholders, such as consumers, in its activities. Stakeholder engagement can serve as a mechanism for fostering cooperation, accountability, trust, and fairness [69]. More precisely, true engagement is built upon an ethical foundation, where interactions between organizations and consumer stakeholders are based on the principles of equity, justice, and fairness [68,70]. In line with this, consumers’ CSR engagement can be viewed as a response to perceived ethicality in organizational practices.

From a communication perspective, the CSR literature defines consumers’ CSR engagement as individuals’ communicative responses to an organization’s CSR efforts, encompassing, but not limited to, “informational seeking, sharing, processing, and commenting behaviors” [71, p. 443]. Scholars primarily view individuals’ engagement in CSR and CSR communication as a critical linchpin in generating desirable outcomes. For example, Park et al. [13] found that employees’ participation in CSR information seeking, updated CSR news browsing, commenting, and supporting behaviors could enhance their own commitment to the organizations and contribute to the increased visibility of their organizations’ CSR communication to external stakeholders. More germane to this study context, previous CSR research has identified that consumers’ CSR engagement is associated with greater support for the organization, including engaging in positive word-of-mouth activities, providing feedback, enhancing brand loyalty, and purchasing products and services from the brand [71–73].

As mentioned earlier, engagement is fostered by a strong ethical foundation, such as fairness [69–70]. Ample research has demonstrated that consumers are more likely to engage in CSR initiatives, information processing, and sharing when they perceive the organization’s efforts as ethical and authentic, rather than as mere profit-driven strategies (e.g., [74–76]). On the contrary, consumers who are skeptical of an organization’s moral standing in running CSR campaigns are less likely to perceive fairness in CSR initiatives, seek and share CSR-related information, or participate in CSR activities [39,71]. Furthermore, skepticism limits consumers’ positive message elaboration and trust-building, which in turn dampens their positive responses, such as attitudes and purchasing decisions, to the organization; in the long term, this skepticism can even erode the potential for relationship-building between the organization and its stakeholders, weakening organizational reputation and brand loyalty [11,40,71,77].

Given the significance of perceived ethics and authenticity in motivating consumer engagement in CSR and the negative effects of CSR skepticism, it is reasonable to hypothesize a negative correlation between CSR skepticism and CSR engagement intention. Thus, we propose the following hypothesis:

**H3**: CSR skepticism is negatively associated with CSR engagement.

### Ethical Purchasing Intention

As a typical manifestation of ethical consumption, ethical purchasing intention refers to an individual’s conscious and reflective buying decisions that align with personal moral beliefs and values, often involving a willingness to pay a premium for products from ethical brands [78,79]. It is a decision that extends beyond economic considerations and reflects consumers’ cognitive and affective evaluations of both the product and the organization behind it. In the context of this study, only when consumers are aware of the ethical standing of an organization’s CSR investments and perceive alignment between their moral standards and the organization’s ethical performance are they likely to purchase from this socially responsible company [30,80].

From the social identity approach, ethical purchasing can be viewed as a means through which consumers express their membership in socially desirable or morally acceptable groups [81]. When individuals define themselves as ethical consumers, they tend to strategically select products that represent their moral identities [82]. Additionally, when consumers’ important social networks expect them to purchase ethical products or support socially responsible brands, they are more likely to do so because people tend to align their behaviors with the values and preferences of their social groups, further reinforcing their social identity [83].

However, as ethical purchasing decisions hinge on consumers’ trust in a brand’s ethical standing, any uncertainty or doubts regarding the organization’s ethicality can diminish their willingness to buy its products. For example, if consumers question the authenticity of the product’s environmental benefits claimed by a green organization, they may refuse to consume the products from the organization and develop distrust toward the organization. This, in turn, increases consumers’ external information-seeking behaviors and unfavorable word-of-mouth communication [84]. Similarly, Kwon and Ahn [85] found that green skeptical customers were less likely to support environmentally friendly hotels. When consumers doubt a company’s commitment to social responsibility, their emotional connection to the brand weakens, making them more susceptible to exploring alternatives that appear more credible and ethically aligned [86].

In sum, when consumers doubt the authenticity or ethical integrity of an organization’s CSR initiatives, their willingness to purchase from the organization significantly decreases as they seek alternatives that align with their moral values. Based on these insights, we propose the following hypothesis:

**H4:** CSR skepticism is negatively associated with ethical purchasing intention.

Prior research has demonstrated the positive effect of consumer CSR engagement on a variety of supportive and prosocial behaviors, including ethical purchasing intentions [73,87,88]. As individuals actively participate in CSR information processing and sharing and make voluntary contributions to CSR initiatives [71], consumer engagement in CSR can foster stronger connections between consumers and organizations. Through the engagement, organizations gain valuable insights into consumers’ expectations of CSR, while consumers develop a deeper understanding of the organization’s benevolence and moral alignments between each other [68]. These positive perceptions may further encourage consumers to identify with the organization and develop ethical purchasing intentions in support of the company [14,89].

Moreover, engaged consumers often invest significant time, emotional energy, and other resources in their interactions with the organization. Such an investment can facilitate a greater personal interest and a stronger bond with the organization, making consumers more likely to identify with it. When consumers identify with socially responsible companies, they are more inclined to take purchase behaviors to express their ethical identity explicitly [81–82]. From a long-term perspective, these consumers are less likely to switch and more likely to advocate for the company and repeatedly purchase from it [72]. Based on these findings, we propose a positive relationship between consumer engagement in CSR and their purchasing intentions as follows:

**H5:** CSR engagement is positively associated with ethical purchasing intention.

## Method

### Data collection

This study was approved by the Institutional Review Board (IRB) of North Carolina State University and was conducted in accordance with relevant ethical guidelines and regulations. The research was considered to pose minimal risk to participants.

Informed consent was obtained from all participants prior to participation. Specifically, participants were presented with an online consent form at the beginning of the survey and indicated their consent before proceeding. They were informed that the study involved a health-related CSR campaign (breast cancer awareness) and that the content was informational in nature. All data underlying the findings of this study are publicly available at https://osf.io/96kja/. The dataset has been fully de-identified to ensure participant anonymity and to minimize any potential risk of re-identification.

Following IRB approval, data collection was conducted through Dynata, a market research company, to obtain a sample that reflected the target population. During the week of June 22–28, 2024, approximately one thousand registered panel participants volunteered to take part in the study. Participants were selected through quota sampling based on gender, age, and income, with quotas aligned with U.S. Census population distributions. This quota sampling approach ensured that the sample matched U.S. Census distributions on selected demographic characteristics (e.g., gender, age, and ethnicity); however, it does not constitute a probability-based sample of the U.S. population. Only U.S. residents were eligible, resulting in 787 valid responses for final analysis. Only fully completed responses that passed all attention-check questions were retained; therefore, there were no missing data in the final analytic sample.

Prior to the main data collection, a pilot test with 100 participants was conducted to evaluate and refine the survey instrument. Open-ended questions were included to capture participants’ perceptions of CSR campaigns and to identify potential issues related to item wording, comprehension, and content validity. Participant feedback from this initial phase was instrumental in improving the survey’s clarity and effectiveness. The pilot data were not included in the final analytic sample and were used solely for questionnaire refinement.

To uphold research integrity, a continuous monitoring process was employed throughout the data collection phase. Several attention-check items were included in the questionnaire to assess respondent attentiveness and engagement. For example, participants were instructed to choose a specific response option, such as “strongly agree,” for designated questions. Those who followed these instructions were deemed qualified to proceed with the survey. To establish a common understanding of the study context, respondents were first introduced to the concept of CSR and informed about the primary objectives of Delta Air Lines’ CSR efforts before proceeding with the survey. This introduction included a brief presentation of Delta’s CSR activities, illustrated with relevant images. These initiatives included aircraft painted pink to promote breast cancer awareness, merchandise sales supporting breast cancer research, a dedicated “Pink Plane” flight recognizing employee breast cancer survivors, and the provision of pink-themed amenities (e.g., napkins and earbuds) to reinforce campaign visibility.

Participants were required to review this information for at least five minutes to ensure adequate familiarity with the campaign. After this introduction, the survey questions explored various aspects of participants’ views, including their ethical judgments, subjective norms, CSR skepticism, engagement, and ethical purchasing intentions. This approach aimed to capture a comprehensive understanding of how Delta’s CSR efforts are perceived and their association with consumer attitudes and responses.

### Common method bias assessment

Given that all variables were measured using self-reported data collected through a single survey, we implemented both procedural and statistical remedies to reduce and assess the potential impact of common method variance (CMV).

Procedurally, a pilot test with 100 participants was conducted to refine the questionnaire and improve item clarity. Based on the pilot feedback, the wording and structure of the survey were revised to reduce ambiguity and potential overlap across constructs. In the main study, participants were assured of anonymity and confidentiality to minimize evaluation apprehension and social desirability bias. In addition, validated scales from prior research were used, and items for different constructs were presented in separate sections to reduce response pattern bias. Attention-check questions were also included to ensure data quality.

Statistically, we assessed CMV using a latent common method factor approach. A common latent factor was added to the measurement model, and the comparison between the baseline model and the model including the common method factor showed no substantial improvement in model fit (ΔCFI < .01; ΔRMSEA < .01). In addition, the inclusion of the common method factor did not materially change the standardized factor loadings, with differences generally below  .10. These results suggest that CMV is unlikely to threaten the validity of the study’s results.

### Sample features

The demographics of the study’s participants provide a comprehensive overview of the sample’s composition. Out of the 787 participants, 46.5% were male and 53.5% were female. The age distribution was diverse, with the largest age group being participants aged 25–44 years, who made up 36.1% of the sample. This was followed by participants aged 65–74, representing 16.7%, and those in the 55–64 age range, who accounted for 14.4%. Individuals aged 45–54 comprised 13.8% of the sample. Participants aged 18–24 constituted 10.2%, while those aged 75 and older represented 8.8%.

The educational background of the study’s participants reveals a diverse range of qualifications. Of the 787 participants, 1.5% had completed less than a high school diploma, while 22.1% had earned a high school diploma or General Educational Development (GED). Participants with some college experience but no degree made up 15.5% of the sample. Those with an associate or technical degree accounted for 13.1%, and 29.6% held a bachelor’s degree. Individuals with a master’s degree constituted 14.4%, and 3.8% had completed a doctorate degree. This distribution shows a broad spectrum of educational attainment, with a significant proportion holding bachelor’s and higher degrees.

The income distribution of the study’s participants provides a clear picture of their financial backgrounds. Out of the 787 participants, 12.8% reported an annual income of $20,000 or under. Those earning between $20,001 and $40,000 made up 18.2% of the sample, while 18.6% earned between $40,001 and $60,000. Participants with incomes ranging from $60,001 to $80,000 accounted for 18.0%. The $80,001 to $100,000 income bracket was represented by 14.2%, and 16.3% had incomes of $100,001 or higher. Additionally, 1.9% preferred not to disclose their income. This distribution highlights a diverse range of income levels, with a notable concentration in the middle-income brackets.

The racial and ethnic composition of the study’s participants is diverse. Out of the 787 respondents, 72.6% identified as Caucasian/White (non-Hispanic), making it the largest group. Black/African American (non-Hispanic) participants comprised 12.5% of the sample. Latino/Hispanic individuals represented 7.6%, and 5.0% identified as Asian/Pacific Islander. Native American/American Indian participants accounted for 1.7%, while 0.8% selected “Other” to describe their racial/ethnic identity.

### Measurement

#### Ethical judgment.

The index for ethical judgment (adopted from Cheng & Shen [62]) was derived from five items measuring perceptions of Delta’s Breast Cancer Awareness CSR activity. Participants rated each item on a scale from 1 (Unethical, Traditionally Unacceptable, Unfair, Unjust, Morally Wrong) to 5 (Ethical, Traditionally Acceptable, Fair, Just, Morally Right). The average of these ratings resulted in an index with high reliability, evidenced by a Cronbach’s alpha of  .94.

#### Subjective norms.

The subjective norms index was computed by averaging responses to four items assessing perceived social support for CSR initiatives. Participants rated each item on a scale from 1 (Strongly Disagree) to 5 (Strongly Agree). This measure showed high reliability with a Cronbach’s alpha of  .83 [35]. The items included, “Most people whose opinions I value would approve of me supporting this CSR campaign,” “Most people who are important to me think that I should support women with breast cancer,” “Most people who are important to me think that I should support companies such as Delta for women with breast cancer,” and “Generally, I want to do what people who are important to me think I should do.”

#### CSR skepticism.

CSR skepticism was measured using four items that evaluate the alignment and authenticity of Delta’s CSR actions. Participants responded on a scale from 1 (Strongly Disagree) to 5 (Strongly Agree). The index, derived by averaging these responses, showed high reliability with a Cronbach’s alpha of  .93. Following the scale from Skarmeas and Leonidou [28], the items included statements such as “Delta’s CSR action is in accordance with the company’s values and beliefs”(reversed), “Delta is being true to itself with its CSR actions” (reversed), “Delta is not standing up for what it believes in,” and “Delta is not a socially responsible company.”

#### CSR engagement.

Engagement with CSR activities was assessed using four items related to participants’ involvement with Delta’s Breast Cancer Awareness campaign. Participants rated their likelihood of engaging in these activities on a scale from 1 (Definitely Will Not) to 5 (Definitely Will). The items (adopted from Cheng et al. [71]) included “I would follow up to find information about Delta’s Breast Cancer Awareness campaign program,” “I would regularly check if there is any new information about Delta’s Breast Cancer Awareness campaign,” “I would volunteer my time for Delta’s CSR campaign,” and “It is likely that I would contribute to the breast cancer cause by getting involved in this CSR campaign.” The average score across these items was used to compute the CSR engagement index, which exhibited high reliability with a Cronbach’s alpha of  .93.

#### Ethical purchasing intention.

Ethical purchasing intention was evaluated through four items concerning participants’ preferences and willingness to pay more for Delta’s products due to its CSR initiatives. Respondents rated each item on a 5-point scale from 1 (Strongly Disagree) to 5 (Strongly Agree). The questions adapted from Magano et al. [79] included “I would prefer to purchase from Delta rather than other brands since it is a socially responsible company,” “After seeing this CSR campaign, I prefer to buy from Delta although others are good as well,” “I would recommend Delta’s products/services to my friends due to its CSR initiatives,” and “I am willing to pay a higher price for Delta’s products/services than for other brands due to its commitment to CSR activities.” Responses were averaged to create a composite index, which exhibited excellent reliability (Cronbach’s α = .91).

#### Control variables.

Consumer engagement and reactions to CSR initiatives may vary according to demographic characteristics, CSR fit, and prior satisfaction with the organization [71,76,90]. To address these potential influences, we included controls for these factors in our analysis. CSR fit was assessed using a scale developed by Park [76]. For example, participants were asked, “How well do Delta’s breast cancer CSR initiatives align with the company’s values?” with responses ranging from “Inconsistent” to “Consistent,” evaluating how well the CSR initiatives match Delta’s products, customer base, and company identity.

Prior satisfaction with Delta Air Lines was measured with items from Chen et al. [90]. A sample question was, “To what extent do you agree with the statement, ‘Both Delta and I benefit from our relationship?’” In addition to these factors, we controlled demographic variables such as education, gender, and age, as these elements could also impact the results of our theoretical model.

## Results

### Descriptive statistics

To categorize the value ranges of the measured variables in this study, we used the following scale points: “low (1.00–1.99),” “moderately low (2.00–2.99),” “neutral (3.00),” “moderately high (3.01–3.99),” and “high (4.00–5.00).” The results of the descriptive analysis revealed the following patterns among participants:

Participants demonstrated a moderately low level of skepticism toward CSR initiatives, with a mean score of 2.20 (SD = .80). In contrast, they exhibited a high level of ethical judgment, reflected by a mean score of 4.21 (SD = .82). Engagement with CSR activities was moderately high, with a mean score of 3.05 (SD = 1.11). Ethical purchasing intention was also moderately high, with a mean score of 3.19 (SD = 1.02). Perceived social support for CSR, measured by subjective norms, was moderately high, with a mean score of 3.69 (SD = .78).

Overall, participants showed moderately low skepticism, high ethical judgment, and moderately high levels of CSR engagement, ethical purchasing intention, and perceived social support for CSR. The correlations among these variables ranged from moderate to strong, indicating meaningful relationships within the model.

### Measurement model

The confirmatory factor analysis (CFA) model demonstrated a strong fit with the data, as evidenced by the following indices: χ^2^ = 487.709, df = 174, χ^2^/df = 2.803, SRMR = .04, RMSEA = .048 (90% CI = .043–.053), CFI = .98, TLI = .97, with a sample size of 787. To assess the reliability and validity of the measures, we computed the average variance extracted (AVE) and composite reliability (CR). As detailed in Table 1, factor loadings ranged from  .63 to  .91; CR values ranged between  .85 and  .94, and AVE values ranged between  .60 and  .76, indicating robust measurement reliability [91].

**Table 1:** Results of the Measurement Model, AVE & CR.

Factors	Indicators	Loadings	AVE&CR
Ethical judgment [62]	The Breast Cancer Awareness CSR activity from Delta to me is: Unethical: Ethical	.86***	AVE = .74 CR = .94
	Traditionally Unacceptable: Traditionally Acceptable	.81***	
	Unfair: Fair	.88***	
	Unjust: Just	.90***	
	Morally wrong: Morally right	.86***	
Subjective norms [35]	Most people whose opinions I value would approve of me supporting this CSR campaign.	.82***	AVE = .60 CR = .85
	Most people who are important to me think that I should support women with breast cancer.	.74***	
	Most people who are important to me think that I should support companies such as Delta for women with breast cancer.	.88***	
	Generally, I want to do what people who are important to me think I should do.	.63***	
CSR Skepticism [28]	Delta’s CSR action is in accordance with the company’s values and beliefs (Reversed).	.86***	AVE = .75 CR = .92
	Delta is being true to itself with its CSR actions (Reversed).	.88***	
	Delta is not standing up for what it believes in.	.87***	
	Delta is not a socially responsible company.	.86***	
CSR Engagement [71]	I would follow up to find information about Delta’s Breast Cancer Awareness campaign program.	.88***	AVE = .76 CR = .93
	I would regularly check if there is any new information about Delta’s Breast Cancer Awareness campaign.	.89***	
	I would volunteer my time for Delta’s CSR campaign.	.83***	
	It is likely that I would contribute to the breast cancer cause by getting involved in this CSR campaign.	.88***	
Ethical purchasing intention [79]	I would prefer to purchase from Delta rather than other brands since it is a socially responsible company.	.91***	AVE = .73 CR = .91
	After seeing this CSR campaign, I prefer to buy from Delta although others are good as well.	.88***	
	I would recommend Delta’s products/services to my friends due to its CSR initiatives.	.82***	
	I am willing to pay a higher price for Delta’s products/services than for other brands due to its commitment to CSR activities.	.79***	

*Note.* χ^2^ = 487.709, df = 174, χ^2^/df = 2.803, SRMR = .04, RMSEA = .048 (90% CI = .043–.053), CFI = .98, TLI = .97, n = 787; AVE = Average Variance Extracted; CR = Composite Reliability; *** p < .001.

Additionally, the CFA and SEM analyses were conducted using maximum likelihood (ML) estimation. All indicators were treated as continuous variables, consistent with prior research using Likert-type scales [92]. Multivariate normality was assessed by examining skewness and kurtosis values. Skewness ranged from −.97 to  .76 and kurtosis ranged from −.66 to 1.16, which fall within acceptable thresholds (|skewness| < 2 and |kurtosis| < 7; [93]), indicating no severe deviation from normality.

Discriminant validity was assessed using multiple approaches. First, following the Fornell–Larcker criterion, the square root of the AVE for each latent construct ranged from  .77 to  .87 and exceeded the corresponding inter-construct correlations (see Table 2). In addition, all inter-construct correlations were below the commonly accepted threshold of  .85 [94], providing further support for construct distinctiveness.

**Table 2:** Correlations Among Variables.

Variables	Ethical Judgment	Subjective Norms	CSR Skepticism	CSR Engagement	Ethical Purchasing Intention
**Ethical Judgment**	**.86**				
**Subjective Norms**	.54**	**.77**			
**CSR Skepticism**	−.59**	−.66**	**.87**		
**CSR Engagement**	.39**	.61**	−.58**	**.87**	
**Ethical purchasing intention**	.45**	.66**	−.65**	.75**	**.85**

Note. **. Correlation is significant at the 0.01 level (2-tailed). Values on the diagonal (bolded) represent the square roots of the average variance extracted (AVE), whereas the off-diagonal values indicate the correlations among constructs.

In addition, the heterotrait–monotrait ratio (HTMT) was examined. All HTMT values were below the conservative threshold of  .85 [95], providing further support for discriminant validity. Furthermore, the average variance extracted (AVE) values ranged from  .60 to  .76, exceeding the recommended threshold of  .50, and composite reliability (CR) values ranged from  .85 to  .94, indicating strong internal consistency [96].

### Results of the structural model

The hypothesized structural model demonstrated good fit with the collected data: χ^2^ = 727.180, df = 274, χ^2^/df = 2.654, SRMR = .04, RMSEA = .046 [90% CI = .042−.050], CFI = .97, TLI = .97, n = 787.

**Fig 2:**
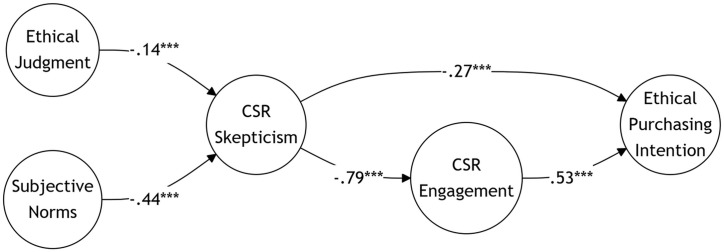
Structural model with results. Note. Control variables: Age, education, gender, CSR fit, and prior satisfaction with Delta. χ^2^ = 727.180, df = 274, χ^2^/df = 2.654, SRMR =.04, RMSEA =.046 [90% CI =  .042−.050], CFI =.97, TLI =.97, n = 787.

**Hypothesis testing.** H1 posits that ethical judgment is negatively associated with CSR skepticism, which was supported by the data (as shown in Fig 2). Analysis revealed a significant negative relationship (β = −.14, p < .001), indicating that higher levels of ethical judgment are negatively associated with skepticism toward CSR initiatives. Participants with a stronger ethical perspective are less likely to question the motives behind CSR activities, suggesting that ethical judgments are pivotal in shaping perceptions of corporate social responsibility.

Similarly, H2, which proposes that subjective norms are negatively associated with CSR skepticism, was confirmed. The results showed a substantial negative effect (β = −.44, p < .001), indicating that when individuals perceive strong social support for CSR initiatives, they are less likely to be skeptical. This underscores the significant role of social context and expectations in mitigating skepticism toward CSR efforts.

H3 hypothesized that CSR skepticism is negatively associated with CSR engagement, which was supported by the findings. Analysis revealed a strong negative relationship (β = −.79, p < .001), indicating that higher skepticism toward CSR initiatives is linked to lower levels of engagement. Participants with more skepticism are less likely to actively support or participate in CSR activities. Similarly, the hypothesis that CSR skepticism is negatively linked to ethical purchasing intention was supported (H4). The results showed a significant negative relationship (β = −.27, p < .001), suggesting that higher levels of skepticism toward CSR are associated with lower ethical purchasing intention. This highlights how skepticism can deter consumers from forming ethical purchasing intention.

Conversely, H5, which assumes that CSR engagement is positively related to ethical purchasing intention, was supported. The analysis demonstrated a significant positive relationship (β = .53, p < .001), indicating that greater engagement with CSR initiatives correlates with a higher likelihood of forming ethical purchasing intention. Participants reporting higher intentions to engage in CSR activities were also more likely to report stronger ethical purchasing intention.

In addition, the effects of control variables are reported in Table 3. The results indicate that CSR fit (β = −.26, p < .001) and prior satisfaction (β = −.18, p < .001) were both negatively associated with CSR skepticism, indicating that higher perceived fit and satisfaction are linked to lower levels of skepticism. In addition, age was negatively associated with CSR engagement (β = −.21, p < .001), suggesting that older participants reported lower levels of engagement. Furthermore, prior satisfaction was positively associated with ethical purchasing intention (β = .23, p < .001), indicating that individuals with more favorable prior experiences with the brand were more likely to report stronger ethical purchasing intention.

**Table 3:** Effects of Control Variables in the Structural Model.

Outcome	Predictor	β	S.E.	C.R.	p
**CSR skepticism**	CSR fit	−.26	.03	−7.56	<.001
	Prior satisfaction	−.18	.02	−6.56	<.001
	Age	−.03	.00	−1.11	.27
	Gender	−.03	.04	−1.20	.23
	Education	.03	.01	1.38	.17
**CSR engagement**	Age	−.21	.00	−6.78	<.001
	Prior satisfaction	.02	.05	.45	.66
	Gender	.02	.07	.58	.56
	Education	.01	.02	.28	.78
	CSR fit	−.05	.06	−.83	.41
**Ethical purchasing intention**	Prior satisfaction	.23	.03	8.74	<.001
	Gender	.04	.04	1.65	.10
	Education	−.00	.01	−.17	.87
	Age	−.04	.00	−1.64	.10
	CSR fit	.04	.03	1.38	.17

**Note**. Only control variable effects are reported. Hypothesized relationships are presented in Fig 2. Standardized coefficients (β) are shown. CSR = corporate social responsibility.

**Indirect effects** Mediation effects were tested using bias-corrected bootstrapping (5,000 resamples). Results shed light on how ethical judgment, subjective norms, and CSR skepticism are related to CSR engagement and ethical purchasing intention. Specifically, CSR skepticism was found to be a significant mediator in several relationships. It mediated the connection between ethical judgment and CSR engagement (β = .11, SE = .03, p < .001, BC 95% CI:  .05 to  .17), as well as between subjective norms and CSR engagement (β = .35, SE = .03, p < .001, BC 95% CI:  .29 to  .42). Additionally, CSR skepticism mediated the relationships between ethical judgment and ethical purchasing intention (β = .09, SE = .03, p < .001, BC 95% CI:  .04 to  .15) and between subjective norms and ethical purchasing intention (β = .31, SE = .03, p < .001, BC 95% CI:  .24 to  .37). Furthermore, CSR engagement mediated the effect of CSR skepticism on ethical purchasing intention (β = −.42, SE = .04, p < .001, BC 95% CI: −.51 to −.35).

## Discussion

In parallel with the flourishing of CSR campaigns, there is an exponential growth in scholarly research on ethical consumption behaviors within the domain of business management because of the importance of ethical consumption on business success and societal sustainability and well-being [49,50]. This study assembles an extended theoretical lens that builds on the TRA framework [17] and extends it by integrating the insights of consumers’ ethical judgment and skepticism toward organizational CSR efforts. In particular, this study examines how ethical judgment and subjective norms are related to consumer skepticism, CSR engagement intention, and ethical purchasing intention in relation to Delta’s Breast Cancer Awareness campaign.

The survey results revealed several key insights. Positive ethical judgments of Delta’s campaign and stronger perceived social expectations to support it were associated with lower levels of skepticism toward the campaign. Lower levels of skepticism are, in turn, associated with higher levels of consumers’ intentions to actively seek campaign-related information, contribute personal resources, and purchase Delta’s products and services. Moreover, those with a higher intention to engage in Delta’s campaign were more willing to consume Delta’s services. In this study, CSR skepticism is examined as a mediating variable between ethical judgment (as well as subjective norms) and ethical behavioral intentions (such as engagement and purchasing). The theoretical and practical implications of these findings, which are discussed in detail below, shed light on the complex dynamics of consumer behavior in the context of CSR initiatives.

### Theoretical and practical implications

The ethical attribution of CSR has been emphasized in the business world [97]. Mitnick et al. [43] argue that an effective CSR campaign should reflect an organization’s “moral purpose of business and its proper relationship to society” (p. 192). However, the research on the ethical component of CSR has primarily been conducted from an organization’s internal perspective, leaving the ethical judgment from the consumers’ perspective under-examined [98].

This study aims to enrich the scholarship of CSR and ethical consumption by investigating consumers’ evaluation of the morality of CSR campaigns and their subsequent reactions. Our findings align with the ethical decision-making literature (e.g., [45]) and provide additional empirical evidence highlighting the important role of ethical judgment in consumers’ ethical consumption decisions in the context of CSR campaigns. This study indicates that consumers who perceive CSR initiatives as morally aligned with their values are associated with a higher likelihood of engagement in CSR activities and stronger ethical purchasing intention. A similar pattern has been identified in the role of subjective norms on consumers’ reactions toward CSR campaigns. The perceived social pressure to consume ethically is further associated with stronger ethical behavioral tendencies, as consumers are more likely to trust and support ethical businesses when they feel that their social networks expect them to do so. Additionally, participants’ CSR engagement intention was positively associated with their purchasing willingness. These findings suggest that as consumers volunteer more resources to the campaign and get to know it better, they are more likely to report stronger purchasing intentions toward the organization [99].

CSR campaigns, however, are not always effective in facilitating ethical consumption, especially when skepticism arises. Echoing the findings from previous research on CSR skepticism (e.g., [23,76]), the study findings provide additional support for the negative association between CSR skepticism and consumer responses. In this study, we pay special attention to the disbelief of an organization’s ethics rooted in the concept of CSR skepticism and adopt an ethical lens to understand consumers’ skepticism.

We argue that CSR campaigns perceived as ethically appropriate and altruistic (i.e., ethical judgment) are associated with lower levels of skepticism. In turn, skepticism, conceptualized as a negative evaluation of a firm’s moral position, is expected to reduce consumer engagement with CSR initiatives and ethical purchasing intention. This proposition is consistent with prior research suggesting that CSR skepticism undermines favorable consumer responses, including purchase intentions and relationship outcomes [28,100]. Thus, we extend the classic TRA model by incorporating CSR skepticism into the model, providing a more comprehensive understanding of ethical consumption behaviors in the context of CSR. The results demonstrate that skepticism, viewed as a negative perception of CSR efforts concerning the moral position of the organization in this study, mediates the relationships between ethical judgment and ethical behavioral intentions and between subjective norms and ethical behavioral intentions.

Additionally, the path coefficients show that subjective norms, compared to ethical assessment, show a stronger association with skepticism in the context of CSR campaigns. Compared to an individual’s internal cognitive mechanism regarding ethics, the surrounding environment and collective opinions show a stronger association with ethical decision-making as long as the environment is vital for the individual. This means that consumers’ perceptions of CSR are not formed in a vacuum but are influenced heavily by the environment, suggesting that consumers’ perceptions and responses are closely related to their social environment.

Given the above, we provide some strategic insights for marketing, advertising, and public relations professionals to run CSR campaigns effectively to foster ethical consumption. First, professionals should be mindful of consumers’ ethical standards when designing CSR campaigns. Campaigns that align with the moral standards of target audiences may be more effective in fostering favorable consumer responses. As individuals may have different understandings and expectations of business ethics in nuance, it will be beneficial for corporations to address social issues perceived as ethically crucial for the majority of the targeted audience and meet the universal moral codes in their CSR campaigns, such as environmental protection and climate change.

Furthermore, as the contextual factor (i.e., social expectations) significantly impacts personal perceptions and behaviors, professionals should be aware of the value of “buzz” in triggering favorable outcomes from their CSR investments. Prior research has shown that both mobile and AI-enabled CSR communication strategies can effectively enhance stakeholder engagement and supportive responses toward organizations [100,101]. Therefore, professionals should engage their consumers through online communities and enhance consumers’ community identification or even brand identification. Once the audience develops a strong sense of belonging to the group, the group’s voices become influential and perceived as social norms that guide their future behaviors. Through encouraging collective opinions, companies may benefit from fostering an environment for their CSR campaign because social expectations are associated with lower levels of skepticism.

### Limitations and suggestions for future research

This study has several limitations. First, prior methodological research has highlighted that SEM-based analyses often rely on strong causal assumptions that are not directly testable, especially when using cross-sectional data [102]. Consistent with this perspective, the findings of this study are interpreted as associations rather than causal effects.

Second, the use of a single CSR campaign context may limit the interpretation of the findings, as participants’ responses may be influenced by preexisting attitudes toward the brand or the campaign topic. To establish a common understanding of the study context, participants were first exposed to explanatory information about CSR and the focal campaign before proceeding with the survey. While this ensured a shared understanding of the context, it may have also introduced a priming effect, potentially leading to more favorable evaluations and lower levels of skepticism. Moreover, as all constructs were measured following exposure to the same campaign stimulus, respondents may have formed closely related perceptions across conceptually similar constructs.

Furthermore, although a relatively strong negative association was observed between CSR skepticism and engagement, this relationship warrants careful interpretation. Future research is encouraged to examine this relationship using alternative model specifications or longitudinal or experimental designs. Finally, the use of quota sampling through an online panel may limit the generalizability of the findings to the broader U.S. population.

## Conclusion

This research integrates individual (i.e., ethical judgment) and contextual factors (i.e., subjective norms) to explore individuals’ reactions toward an organization’s CSR efforts. By incorporating the role of CSR skepticism in consumers’ ethical consumption decisions, this study provides a more comprehensive framework to understand the ethical decision-making process. The findings from this research indicate that both ethical judgment and subjective norms are significantly associated with consumers’ negative perceptions of CSR initiatives (i.e., skepticism), with perceived social approval of CSR showing a stronger association with skepticism. Lower levels of skepticism are associated with higher levels of CSR engagement and ethical purchasing intention. Professionals are advised to select CSR issues that resonate with the ethical feelings of the targeted audience for their CSR campaigns. Professionals should also consider strategies that foster supportive social norms when launching CSR campaigns.
